# Upadacitinib in rheumatoid arthritis: progress and challenges

**DOI:** 10.3389/fphar.2026.1776317

**Published:** 2026-03-17

**Authors:** Qiaofen Wang, Long Zhong, Yujing Fu, Zhou Yang

**Affiliations:** 1 Department of Medicine, Hainan Medical University, Haikou, China; 2 Department of Rheumatology and Immunology, Hainan Affiliated Hospital of Hainan Medical University (Hainan General Hospital), Haikou, China; 3 Shenzhen University Medical School, Shenzhen University, Shenzhen, Guangdong, China

**Keywords:** cost-effectiveness, JAK inhibitors, precision medicine, rheumatoid arthritis, safety, upadacitinib

## Abstract

Rheumatoid arthritis (RA) is a chronic autoimmune disease characterized by progressive joint destruction and systemic inflammation, imposing a substantial global health burden, with an estimated 31.7 million cases projected by 2050. Despite advances in therapy, limitations of conventional synthetic disease-modifying antirheumatic drugs (csDMARDs) and biologic DMARDs (bDMARDs) persist, including adverse effects, high costs, and inadequate responses in certain patient subgroups. Upadacitinib (UPA), a selective Janus kinase (JAK) 1 inhibitor, represents a breakthrough in targeted synthetic DMARDs (tsDMARDs), offering rapid symptom relief and structural preservation. Clinical trials, including the SELECT-COMPARE study, have demonstrated that UPA outperforms adalimumab in achieving higher 12-week American College of Rheumatology (ACR) 20 (71% vs. 63%), ACR50 (45% vs. 29%), and ACR70 responses, with sustained efficacy extending beyond 5 years. Real-world evidence from the Canadian post-marketing observational CLOSE-UP study corroborated these findings, showing that 63.5% of patients achieved DAS28-CRP ≤2.6 at 6 months. Compared with pan-JAK inhibitors such as tofacitinib, the JAK1 selectivity of UPA may reduce off-target effects; however, safety concerns remain, including an increased risk of herpes zoster (particularly in Asian populations), liver enzyme abnormalities, and potential cardiovascular events. Cost remains a major barrier, with annual expenses exceeding USD 60,000 for uninsured patients, although partially alleviated by insurance coverage. Future directions highlight combination strategies, personalized treatment approaches, and broader applications in psoriatic arthritis and ankylosing spondylitis. While UPA enhances RA management through its oral convenience and robust efficacy, addressing long-term safety monitoring, affordability, and mechanisms underlying refractory RA remains imperative for optimizing global patient outcomes.

## 1 Introduction

### 1.1 Background of rheumatoid arthritis

Rheumatoid Arthritis (RA) is a chronic autoimmune disease characterized primarily by erosive arthritis, often leading to joint deformities and functional impairment. It presents as symmetric polyarthritis and can affect both large and small joints. RA may result in damage to joints and surrounding soft tissue structures, as well as systemic inflammatory consequences. The disease is highly disabling, and if left untreated, the chronic inflammation of the synovium can lead to severe joint damage, disability, and loss of work capacity as the disease progresses (Smolen et al., 2016; McInnes and Schett, 2011; Firestein, 2003). Currently, there is no cure for RA, and the main therapeutic goal is to control inflammation early while preserving joint structure and function. Early diagnosis is critically important for treatment and prognosis, as it can prevent or significantly slow the progression of joint damage in up to 90% of patients (Aletaha and Smolen, 2018).

Currently, the drugs used in clinical practice mainly include non-steroidal anti-inflammatory drugs (NSAIDs), glucocorticoids, and disease-modifying anti-rheumatic drugs (DMARDs). DMARDs include conventional synthetic DMARDs (csDMARDs), targeted synthetic DMARDs (tsDMARDs), and biological DMARDs (bDMARDs) (Smolen et al., 2016; Firestein and McInnes, 2017).

**FIGURE 1:**
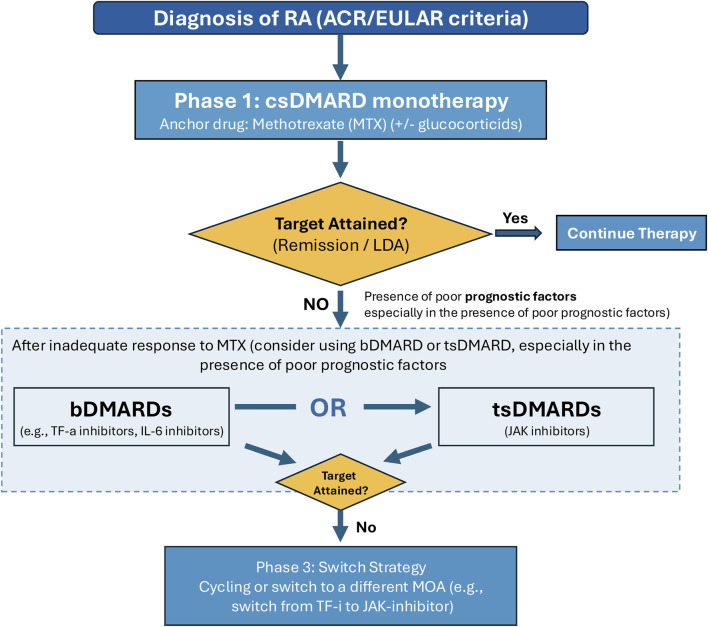
Treatment algorithm for Rheumatoid Arthritis. Note: The schematic shows the progression from diagnosis to treatment with csDMARDs (e.g., Methotrexate). Upon inadequate response, treatment escalates to bDMARDs (e.g., TNF-α inhibitors) or tsDMARDs (e.g., Upadacitinib), in accordance with ACR and EULAR guidelines.

According to the 2021 American College of Rheumatology (ACR) Guideline and the 2022 EULAR recommendations for the management of RA, treatment should typically initiate with csDMARDs (Smolen et al., 2023; Fraenkel et al., 2021). Among them, Methotrexate (MTX) remains the anchor drug and is approved by both the U.S. Food and Drug Administration (FDA) and the European Medicines Agency (EMA) as a first-line therapy (Smolen et al., 2023; Fraenkel et al., 2021). For patients with an inadequate response to csDMARDs, current guidelines recommend stratification to advanced therapies (Smolen et al., 2023; Fraenkel et al., 2021). These include bDMARDs (such as the FDA/EMA-approved TNF-α inhibitors adalimumab and etanercept, and IL-6 inhibitors like tocilizumab) and tsDMARDs. Specifically, Upadacitinib (UPA), along with other JAK inhibitors like tofacitinib and baricitinib, has received regulatory approval from both the FDA and EMA for the treatment of moderate-to-severe active RA, offering a crucial oral therapeutic alternative for refractory patients (Smolen et al., 2023; Burmester et al., 2018) (Figure 1).

Clinical trials have demonstrated that these drugs can effectively improve the condition of RA patients. CsDMARDs, with MTX as the representative, are the first-line drugs for treating RA and are highly effective. Combining with short-term glucocorticoids can rapidly control inflammation, and long-term use can delay joint destruction. However, when using MTX and other csDMARDs, several side effects are commonly reported, including gastrointestinal reactions (nausea, mouth ulcers), hepatotoxicity, and bone marrow suppression (requiring folic acid supplementation for prevention). It is estimated that over 40% of patients receiving MTX are affected, and all patients require frequent monitoring for cytopenia and liver enzyme abnormalities. Up to one-third of patients discontinue MTX treatment within the first year due to various reasons (Wang et al., 2018; Singh et al., 2016). Other csDMARDs (such as leflunomide) have weaker efficacy, and some patients have poor tolerance, making it difficult to meet the treatment needs of patients.

Compared to csDMARDs, the emergence and availability of bDMARDs represent a significant milestone in the history of RA treatment. bDMARDs primarily target inflammatory cytokines or regulate the activation of immune cells involved in the disease’s progression. These include tumor necrosis factor-alpha (TNF-α) inhibitors (such as infliximab, golimumab, etanercept, adalimumab, and certolizumab pegol), interleukin-6 (IL-6) inhibitors (such as tocilizumab and sarilumab), T-cell modulators (such as abatacept), and B-cell modulators (such as rituximab (RTX)) (Bullock et al., 2018).

TNF-α inhibitors are well-established and widely used bDMARDs for the treatment of RA, particularly effective in patients who have inadequate responses to or are intolerant of MTX, and can significantly reduce disease activity (Losina and Katz, 2017). However, they are expensive and are associated with potential adverse effects, including an increased risk of major adverse cardiovascular events (MACE) (Mattay et al., 2024), exacerbation of heart failure (Hussain et al., 2021), and heightened risks of hepatitis virus, tuberculosis infection, and reactivation (Gallowayet al., 2010).

IL-6 inhibitors (such as tocilizumab) are suitable for patients with inadequate responses to TNF inhibitors. Common adverse effects include, diverticulitis, cytopenias, infections, gastrointestinal discomfort, rashes, headaches, and abnormalities in lipid levels or liver enzymes (Jones et al., 2010).

Given the central role of T cells in autoimmunity (Marson et al., 2015; Jeffrey et al., 2015), the T-cell modulator abatacept is effective for treatment-resistant RA in patients with TNF inhibitor resistance. However, its long-term ability to maintain remission or delay joint damage remains unclear (Nakachi et al., 2017; Smolen et al., 2010).

The B-cell depleting agent RTX supports the role of B cells in the pathogenesis of RA (Barnas et al., 2019), When used in combination with MTX or cyclophosphamide (CYC), RTX demonstrates significantly superior efficacy compared to MTX monotherapy, but RTX monotherapy has not shown this advantage (Kaegi et al., 2019).

In recent years, significant advances in the treatment of RA have led to the development of tsDMARDs, such as Janus kinase (JAK) inhibitors. These drugs play a role in blocking the inflammatory process. Multiple clinical studies have shown that JAK inhibitors have comparable efficacy and safety to tumor necrosis factor inhibitors (TNFi). Additionally, the introduction of JAK inhibitors has greatly improved adherence among RA patients. These drugs provide an alternative for patients who have inadequate responses to csDMARDs or bDMARDs.

**FIGURE 2:**
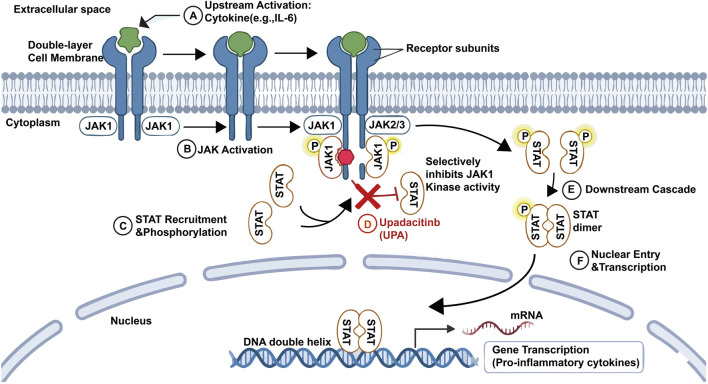
The JAK-STAT signaling pathway and the mechanism of action of Upadacitinib. Note:The figure illustrates the binding of cytokines to cell surface receptors, leading to receptor dimerization and JAK activation. Activated JAKs phosphorylate STAT proteins, which then dimerize and translocate to the nucleus to regulate gene transcription. Upadacitinib selectively inhibits JAK1, thereby blocking the downstream signaling of pro-inflammatory cytokines such as IL-6 and IFN-γ.

The primary representative drugs of tsDMARDs are JAK inhibitors, such as tofacitinib, baricitinib, UPA, and filgotinib, which treat RA by blocking the JAK-STAT signaling pathway. The JAK-STAT signaling pathway consists of three components: JAK-associated receptors, JAKs, and STATs. The signaling mechanism involves the binding of extracellular ligands to receptors on the cell membrane, leading to receptor dimerization. Subsequently, the ligand-receptor complex recruits JAKs intracellularly, where JAKs become activated through tyrosine phosphorylation. The activated JAKs catalyze the phosphorylation of tyrosine residues on the receptor, recruiting STAT proteins with SH2 domains. JAK then catalyzes the phosphorylation of STAT proteins, and the activated STAT proteins dimerize and enter the cell nucleus, where they bind to target genes and regulate gene transcription. This process transmits cellular signals from the extracellular environment to the cell, completing signal transduction and playing a role in regulating immunity, development, disease mechanisms, and cellular homeostasis (Kiełbowski et al., 2024; O'Shea et al., 2013; Leonard et al., 1998) (Figure 2).

The progression of RA is associated with various immune cells (such as T lymphocytes, B lymphocytes, and macrophages) and the synovial inflammation mediated by corresponding cytokines. Cytokines have specific effects on cell-to-cell interactions and communication, including IL-2, IL-3, IL-4, IL-5, IL-6, IL-7, interferons, growth hormone, epidermal growth factor, and others (You et al., 2020). These cytokines can transmit signals through the JAK-STAT signaling pathway, thereby promoting inflammation and joint destruction, leading to the development and progression of RA. Inflammation and immune responses mediated by the JAK-STAT signaling pathway are characterized by elevated expression levels of pro-inflammatory cytokines such as IL-6, IL-2, IL-15, IL-21, and IFN-γ. These cytokines promote inflammation and autoimmune responses through the JAK-STAT pathway, which are key factors in causing joint damage and systemic symptoms. Targeting this pathway with JAK inhibitors has become an important strategy in RA treatment, gradually changing traditional therapeutic approaches (O'Shea et al., 2015; Schwartz et al., 2017).

JAK inhibitors include both selective and non-selective types. However, non-selective JAK inhibitors are associated with a broader range of side effects, as they affect multiple cytokine signaling pathways. Non-selective JAK inhibitors simultaneously inhibit JAK1, JAK2, JAK3, Tyrosine Kinase 2 (TYK2)., which may impair the body’s immune surveillance function, thereby increasing the risk of bacterial, fungal, and viral infections (such as herpes zoster and tuberculosis) (Cohen et al., 2017). Some studies also suggest that the use of non-selective JAK inhibitors may lead to elevated Low Density Lipoprotein (LDL) and High Density Lipoprotein (HDL) levels, increasing the risk of cardiovascular events. This suggests that these drugs may be associated with a higher incidence of cardiovascular events (such as myocardial infarction and stroke) in certain high-risk populations (Ytterberg et al., 2022), along with other side effects. These drawbacks necessitate a careful balance between efficacy and safety when applying these drugs in clinical practice, particularly in high-risk patients (such as the elderly, those with a history of infections, or those with cardiovascular disease), where more refined individualized treatment strategies and monitoring are often required.

With the continuous development and introduction of novel JAK inhibitors, selective JAK inhibitors have gradually emerged as having distinct advantages. By binding to different receptors, they pair with intracellular dimerized cytokine receptor chains, thereby reducing inhibition of non-disease-related cytokine signaling pathways and minimizing off-target effects (O'Shea and Plenge, 2012; Choy, 2019; T Virtanen et al., 2019).

RA is a complex chronic autoimmune disease. Although traditional treatment methods have certain efficacy, some patients exhibit poor responses to these therapies. With in-depth research into the pathogenesis of RA and the continuous emergence of novel targeted therapies, precision medicine has become the future direction of treatment. The research progress of UPA provides an important reference for achieving precision treatment in RA. UPA, as a selective JAK1 inhibitor, precisely targets the JAK1 signaling pathway, blocking the signaling of pro-inflammatory cytokines (such as IL-6, IL-23, etc.), thereby inhibiting the inflammatory response while reducing adverse effects and improving patient adherence.

This review aims to summarize the current progress of UPA in RA, focusing on its clinical efficacy, safety, challenges, and future research directions.

### 1.2 Development and regulatory milestones

The development of UPA was driven by the therapeutic need for safer, more targeted inhibition within the JAK family. In early *in vitro* characterization, UPA demonstrated a distinct pharmacological profile with a 40-fold, 130-fold, and 190-fold higher selectivity for JAK1 over JAK2, JAK3, and TYK2, respectively (Parmentier et al., 2018). This high selectivity was designed to maximize therapeutic efficacy while mitigating off-target effects associated with pan-JAK inhibition.

The clinical validation of UPA was established through the extensive SELECT Phase 3 program. Key trials, such as SELECT-COMPARE, demonstrated that UPA combined with methotrexate (MTX) achieved superior clinical and functional outcomes compared to both placebo and adalimumab. Similarly, in the SELECT-MONOTHERAPY study, UPA monotherapy demonstrated superior efficacy compared to MTX in patients with an inadequate response to prior MTX treatment (Smolen et al., 2019).

Based on these robust data, UPA received its first major regulatory approvals in 2019 from both the U.S. FDA and the EMA for the treatment of adult patients with moderately to severely active RA. Following its success in RA, the approval scope of UPA rapidly expanded. In 2022, indications were extended to include Atopic Dermatitis, supported by data showing high efficacy in skin clearance and itch reduction 6, as well as approvals for Psoriatic Arthritis and Ankylosing Spondylitis (AS) (van der Heijde et al., 2019a; van der Heijde et al., 2022).

However, the rapid uptake of JAK inhibitors also prompted rigorous safety scrutiny. Regulatory bodies, including the FDA and EMA, have implemented safety milestones such as boxed warnings regarding the increased risk of serious infections, MACE, and malignancies9. While global Phase 3 trials for AS and other conditions showed favorable safety profiles with no new safety signals (van der Heijde et al., 2019a), real-world evidence continues to monitor these risks, particularly herpes zoster and venous thromboembolism (Burmester et al., 2023).

## 2 Current progress of upadacitinib

### 2.1 Short-term efficacy

In a 12-week SELECT-COMPARE RA study, UPA combined with MTX showed superior clinical and functional outcomes (such as remission rates, low disease activity, physical function, and pain severity) compared to placebo and adalimumab in statistical terms (Fleischmann et al., 2022). There were no significant differences between UPA monotherapy and UPA + MTX for the different activity indices (Witte et al., 2024). However, in patients who were newly treated with MTX or had insufficient response to MTX, monotherapy with (UPA) showed superior efficacy compared to MTX (Josef et al., 2025). Comparing the ACR20, ACR50, and ACR70 responses across groups, by week 12, 71% of patients receiving UPA had an ACR20 improvement response rate, significantly higher than the 36% in the placebo group and 63% in the adalimumab group. UPA was also significantly superior to both placebo and adalimumab in ACR50 and ACR70 response rates, with the ACR50 response rate being 45% in the UPA group, 15% in the placebo group, and 29% in the adalimumab group (Fleischmann et al., 2019). In a clinical trial involving 648 patients with inadequate response to MTX, patients were randomized to continue MTX treatment, or receive 15 mg UPA or 30 mg UPA monotherapy, with the goal of assessing the efficacy differences in achieving ACR20 remission and Disease Activity Score (DAS) 28 (C-reactive protein,CRP) ≤ 3.2 by week 14. Results showed that among the 216 patients in the MTX group, 89 (41%, 95% CI 35–48) achieved ACR20 remission, while 147 (68%) of the 217 patients in the 15 mg UPA group and 153 (71%) of the 215 patients in the 30 mg UPA group reached this standard. In terms of DAS28 (CRP), only 42 patients (19%) in the MTX group achieved ≤3.2, while 97 (45%) patients in the 15 mg UPA group and 114 (53%) in the 30 mg UPA group met this threshold (Smolen et al., 2019). The study data demonstrate that both doses of UPA significantly outperform continuous MTX treatment in improving ACR20 remission rates and lowering DAS28 (CRP) levels. In a phase 3 randomized controlled trial involving 498 patients, participants were randomly assigned to the 15 mg UPA group, 30 mg UPA group, or placebo group. In the primary efficacy endpoint evaluation, the ACR20 response rate was 65% in the 15 mg UPA group, 56% in the 30 mg UPA group, both significantly higher than the 28% in the placebo group (p < 0.0001). In terms of the achievement of DAS28(CRP) ≤ 3.2, the 15 mg UPA group reached 43%, the 30 mg UPA group reached 42%, both significantly superior to the 14% in the placebo group (p < 0.0001) (Genovese et al., 2018). These data suggest that UPA, at both 15mg and 30 mg doses, demonstrates significant therapeutic effects and is superior to placebo in improving clinical symptoms and disease activity in RA patients (Burmester et al., 2018). For patients with RA who had previously responded poorly to biologic agents, UPA outperformed abatacept at week 12 in terms of improving disease activity (average change in DAS28-CRP -2.52 vs. −2.00) and achieving clinical remission (30.0% vs. 13.3%) (Rubbert-Roth et al., 2020). Therefore, multiple clinical trials indicate that UPA shows significantly higher short-term efficacy in improving RA compared to other drugs.

### 2.2 Long-term safety and efficacy

The long-term safety of UPA can be assessed through treatment-emergent adverse events (TEAEs), while its efficacy can be analyzed using randomized groups (non-responder imputation, NRI) or treatment sequences (such as observation). Regarding safety, over a 5-year treatment period, patients receiving UPA treatment showed similar overall rates of any TEAE and serious TEAEs compared to those receiving adalimumab treatment (Rubbert-Roth et al., 2020; Fleischmann et al., 2024).

Its efficacy over more than 5 years, according to the Clinical Disease Activity Index (CDAI) criteria (CDAI ≤10 and CDAI ≤2.8) for low disease activity (LDA) and remission, as well as the proportion of patients achieving DAS28 (CRP) ≤ 3.2 and DAS28 (CRP) < 2.6, was compared between patients randomized to UPA and adalimumab. Through NRI analysis, the clinical remission rate of UPA was consistently higher than that of adalimumab. The efficacy was also analyzed by summarizing treatment sequences. In patients receiving continuous UPA versus continuous adalimumab, the ACR20 response rate remained similar over 5 years, while the ACR50 and ACR70 response rates were typically higher in patients continuously treated with UPA compared to those on continuous adalimumab. In conclusion, UPA showed similar safety to adalimumab over 5 years of long-term treatment, with better efficacy in long-term RA treatment (Fleischmann et al., 2024). In ankylosing spondylitis (AS) treatment, the safety profile of UPA over 3.3 years showed a low incidence of adverse events, with no reports of serious infections (Burmester et al., 2023). In the global Phase III clinical trials for UPA in AS and nr-axSpA indications, no tuberculosis infections were observed (van der Heijde et al., 2019a; Deodhar et al., 2022). Similarly, compared to MTX, UPA demonstrated superior clinical response in RA patients over the entire 5-year trial period. The incidence of adverse events was higher in the UPA 30 mg group, particularly for serious infections, Herpes zoster, and elevated CPK levels. For patients previously untreated with MTX, UPA 15 mg as monotherapy showed better long-term efficacy and an overall favorable risk-benefit profile. These findings support the approval of the 15 mg daily dose by the U.S. FDA and the EMA (van Vollenhoven et al., 2024). UPA demonstrated a higher persistence rate compared with other drugs. There was no difference in response between monotherapy and combination with scDMARDs in all treatment lines; it was even higher in the early treatment lines (in terms of persistence rate) (Scheepers et al., 2024). Additionally, in the Canadian real-world post-marketing observational study (CLOSE-UP) of adult RA patients treated with 15 mg UPA once daily, the 6-month follow-up results showed that 63.5% of patients achieved the DAS28-CRP ≤2.6 (low disease activity) target, and 77.1% reached the DAS28-CRP ≤3.2 target (Bessette et al., 2024). The mid-term analysis of this study indicated that the efficacy of UPA in the real world was consistent with clinical trial data, regardless of whether patients had previously received bDMARDs or tsDMARDs. Even in patients previously exposed to JAK inhibitors, UPA still demonstrated good efficacy (Rubbert-Roth et al., 2020). Meanwhile, studies have shown that switching from anti-TNF treatment to UPA is superior to (similar) cycling, although these results should be treated with caution (Caporali et al., 2024). The safety study of UPA showed that the incidence of TEAEs was 50.3%, with severe adverse events occurring in less than 5% of cases, consistent with previous reports. No new safety signals were identified, and no severe adverse events clearly related to treatment were observed. Preliminary analysis of this study confirmed UPA’s safety and efficacy in the real world (Conaghan et al., 2021).

### 2.3 Comparison with other JAK inhibitors

Currently, the JAK inhibitors approved for clinical use include tofacitinib, baricitinib, UPA, filgotinib, and peficitinib. Peficitinib is only approved for use in Japan, South Korea, and Taiwan. Therefore, peficitinib will not be further discussed in this review.

Tofacitinib is a pan-JAK inhibitor with higher selectivity for JAK1/JAK3 and mild activity against JAK2 and TYK2 (Dhillon, 2017). It was the first small molecule JAK inhibitor approved by the U.S. FDA in 2012 for the treatment of RA. The most common adverse reactions associated with tofacitinib include dizziness, headache, gastrointestinal issues (such as nausea and diarrhea), nasopharyngitis, infections (especially respiratory and urinary tract infections), as well as increased levels of neutrophils, low-density and high-density lipoproteins, and cholesterol. Among these, gastrointestinal disturbances and infections are the most frequently reported (Dikranian et al., 2022). Baricitinib preferentially inhibits JAK1 and JAK2, with low inhibitory activity against JAK3 (Al-Salama and Scott, 2018). It is the second JAK inhibitor approved by the European League Against Rheumatism (EULAR) for the treatment of RA. Baricitinib can be used as monotherapy or in combination with MTX for the treatment of moderate to severe active RA in patients who have inadequate response to or intolerance to ≥1 DMARD. Analysis of multiple prior studies has shown that, in addition to reducing disease activity, baricitinib also has a bone-protective effect, enhancing the mineralization capacity of osteoblasts (Adam et al., 2020), thus inhibiting the radiographic progression of structural joint damage (van der Heijde et al., 2019b). Several randomized controlled trials and meta-analyses have reported that the most common adverse drug reactions are upper respiratory tract infections, increased LDL cholesterol, nausea, and thrombocytosis (Cohen et al., 2017; Taylor et al., 2017). Filgotinib is a highly selective JAK1 inhibitor with substantially greater affinity for JAK1 than for JAK2, JAK3, or TYK2 (Dhillon and Keam, 2020). It was approved in 2020 by the EMA) and in Japan, either as monotherapy or in combination with methotrexate (MTX), for the treatment of moderate to severe active RA in patients with an inadequate response or intolerance to one or more DMARDs (Combe et al., 2021). Owing to its high selectivity for JAK1, Filgotinib theoretically exerts weaker inhibitory effects on JAK2 (involved in hematopoiesis and red blood cell regulation) and JAK3 (related to lymphocyte function). Consequently, in phase III clinical trials such as the FINCH studies, Filgotinib demonstrated minimal effects on hematologic parameters, including anemia, neutropenia, and lymphopenia (Dhillon and Keam, 2020; Genovese et al., 2019). The most common adverse events include nausea, upper respiratory tract infection, urinary tract infection, and dizziness. Similar to other JAK inhibitors, the potential risks of serious infections—particularly herpes zoster—and venous thromboembolism (VTE) require continued vigilance (Winthrop et al., 2022) (Table 1).

**TABLE 1:** Comparison with JAK inhibitors.

Drug	Primary mechanism	Key academic characteristics
Tofacitinib	Pan-JAK (JAK1/JAK3)	First-in-class oral DMARD; established efficacy, though associated with broad JAK inhibition profile
Baricitinib	JAK1/JAK2	Demonstrates efficacy in RA plus a distinct bone-protective effect (enhancing osteoblast mineralization) leading to inhibition of radiographic progression
Filgotinib	Highly selective JAK1	Exhibits a favorable hematologic safety profile (minimal anemia/neutropenia) attributed to high JAK1 selectivity over JAK2
Upadacitinib	Highly selective JAK1	Shows superior clinical efficacy (Remission/LDA) and drug persistence in network meta-analyses and real-world studies versus comparators (other JAKi/TNF-i)

UPA is a novel selective JAK inhibitor that specifically targets the JAK1 pathway. It was approved by the U.S. In cell line experiments designed to assess the cellular activity and selectivity of UPA against individual kinases, UPA demonstrated a 40-fold, 130-fold, and 190-fold higher selectivity for JAK1 over JAK2, JAK3, and TYK2, respectively (Parmentier et al., 2018). Therefore, the higher specificity of JAK inhibition relative to other subtypes may reduce dose-related toxicity and side effects (Taldaev et al., 2021), without significantly compromising efficacy. Currently, It is worth noting that Upadacitinib (Rinvoq) has received FDA and EMA approvals across multiple immune-mediated inflammatory diseases, including Rheumatoid Arthritis, Psoriatic Arthritis, Ankylosing Spondylitis, and Crohn’s Disease (van der Heijde et al., 2019a; Burmester et al., 2023; McInnes et al., 2021). Specifically, for RA, UPA has been approved for the treatment of adults with moderate to severe active RA who have had an inadequate response to MTX. Although RA and Crohn’s Disease affect different organ systems, they share overlapping pathogenic mechanisms driven by dysregulated cytokine responses (Mohamed et al., 2024; Salas et al., 2020). Specifically, the JAK1 pathway mediates the signaling of key pro-inflammatory cytokines such as IL-6, IL-23, and interferons (Mohamed et al., 2024), which are pivotal in the pathogenesis of both synovial inflammation in RA and intestinal inflammation in Crohn’s Disease (Loftus et al., 2023). By selectively inhibiting JAK1, Upadacitinib effectively disrupts these shared inflammatory cascades (Mohamed et al., 2024), providing the mechanistic rationale for its cross-disciplinary efficacy (Mohamed et al., 2024; Loftus et al., 2023). It has shown superior monotherapy efficacy compared to MTX in both MTX-naive and MTX-inadequate responders (Smolen et al., 2019).

Comparative analyses across multiple studies have demonstrated that UPA exhibits superior clinical efficacy and treatment persistence compared with Tofacitinib and other JAK inhibitors in the management of RA. A matching-adjusted indirect comparison (MAIC) by Edwards et al. (2021) revealed that UPA outperformed Tofacitinib in clinical and functional outcomes, including higher ACR response rates and greater improvement in DAS28-CRP scores (Edwards et al., 2021). Network meta-analyses focusing on DMARD-naïve patients (Sung and Lee, 2021; Lee and Song, 2023) further confirmed that UPA had the highest probability of achieving remission and low disease activity (LDA), ranking first in ACR20/50/70 response rates. Real-world evidence also supports this advantage: Youssef et al. (2025) found that UPA showed higher drug persistence and better disease control compared with other JAK inhibitors and TNF inhibitors (Youssef et al., 2025); similarly, Ciciriello et al. (2024) reported a continued increase in UPA use, with a higher proportion of patients switching from other JAK inhibitors to UPA (Ciciriello et al., 2024). In addition, Bergman et al. (2021) demonstrated that UPA led to greater improvements in quality of life (QoL) than Tofacitinib and methotrexate (MTX), along with significant economic benefits, including increased productivity and reduced indirect costs (Bergman et al., 2021).

According to the latest results from the SELECT-COMPARE Phase III study presented at the 2024 EULAR Congress, along with previous data, UPA monotherapy demonstrated outstanding efficacy over a 5-year follow-up period, with a favorable safety profile (van Vollenhoven et al., 2024). In real-world studies, UPA continues to show excellent clinical application potential and remains an effective treatment option for RA.

## 3 Challenges of upadacitinib

### 3.1 Safety issues

The safety of a drug is directly related to the health and life safety of the patient. In clinical practice, any drug must undergo rigorous safety evaluation to ensure that it does not introduce unacceptable risks while treating the disease.

JAK inhibitors exert anti-inflammatory effects by inhibiting the activity of JAK proteins, thereby modulating cytokine signaling pathways. However, this mechanism may also impact other functions of the immune system, leading to potential adverse events. Previous studies have shown that UPA may present certain safety concerns, with common adverse events including nasopharyngitis, acne, headache, and upper respiratory tract infections. Special attention should be given to potential adverse events such as herpes zoster infection, liver dysfunction, opportunistic infections, and cardiovascular events (Schwartz et al., 2017; Fleischmann et al., 2019).

Regarding the safety of UPA during pregnancy, as of April 2023, a total of 128 pregnancy cases with known outcomes involving maternal exposure to UPA had been reported from clinical trials and post-marketing data. The observed adverse pregnancy outcomes—such as spontaneous abortion rates (24% in clinical trials and 38% in post-marketing reports)—were comparable to the background incidence in the general population and in patients with autoimmune diseases. Most importantly, this analysis found no evidence of a teratogenic effect (i.e., causing birth defects) associated with early pregnancy exposure to UPA. Nevertheless, given the limited amount of available data, definitive conclusions regarding the safety of UPA use during pregnancy cannot yet be established (Mahadevan et al., 2024).

#### 3.1.1 Analysis of adverse events

In clinical trials of UPA, it has demonstrated excellent potential in RA; however, some adverse events have been observed in its clinical use, primarily including herpes zoster infection, elevated liver enzymes, and lymphocytopenia (Szekanecz et al., 2024). The incidence of herpes zoster infection was significantly higher in the UPA group compared to the placebo group (Fleischmann et al., 2019; Cohen et al., 2021). For instance, in a Phase III trial of atopic dermatitis patients, the incidence of herpes zoster in the UPA 30 mg group was relatively higher. UPA suppresses the JAK1/STAT pathway, which reduces immune surveillance, leading to the reactivation of latent herpesviruses (such as varicella-zoster virus). If herpes zoster occurs, it is recommended to suspend treatment until symptoms resolve and to administer antiviral therapy (e.g., acyclovir) (Fleischmann et al., 2022). Meanwhile, during UPA treatment, approximately 10%–15% of patients may experience an increase in liver enzymes (Alanine Aminotransferase/Aspartate Aminotransferase, ALT/AST), typically mild to moderate (grade 1–2), although the incidence of severe increases (≥3 times the upper limit of normal) is around 1%–3%. This may be related to the drug’s direct hepatic toxicity or immune-mediated hepatocellular damage (Fleischmann et al., 2022). Lastly, UPA can cause a decrease in lymphocyte count, particularly more pronounced in the 30 mg dose group. For example, in RA trials, about 5% of patients experienced lymphocytopenia (<500 cells/mm^3^). JAK1 inhibition may affect lymphocyte differentiation and survival, leading to a reduction in peripheral blood lymphocytes (Fleischmann et al., 2019).

#### 3.1.2 Long-term cardiovascular risk and potential association with malignant tumors

Long-term use of UPA may increase the risk of MACE, such as myocardial infarction, stroke, and cardiovascular death (expanding the cardiovascular risk ratio) (Kameda et al., 2021). In the long-term extension studies of UPA, the risk was more pronounced in high-risk populations, such as those aged ≥50 years, smokers, and individuals with diabetes. JAK inhibitors may increase the overall incidence of malignancies. The risk of malignancies has been widely noted in clinical trials and long-term follow-up studies of JAK inhibitors (Pippis and Yacyshyn, 2020). In UPA clinical trials, a few cases of malignancies were reported. In one clinical trial, non-melanoma skin cancer (NMSC) was the most frequently reported malignancy in both treatment groups, with only 4 cases; aside from NMSC, no specific types or patterns of malignancies were observed (Simpson et al., 2022).

### 3.2 Drug resistance and treatment resistance

In the treatment of RA, the resistance rate to UPA ranges from 5% to 30% (Burmester et al., 2018; Sanmartí and Corominas, 2023). According to clinical research data, approximately 5%–20% of RA patients are unresponsive to all current medications (including biologics and targeted therapies), leading to joint damage, disease progression, multisystem complications, and other adverse prognoses (Nagy et al., 2020; de Hair et al., 2018).

### 3.3 Cost and accessibility

Although UPA has demonstrated superior efficacy in rapidly alleviating symptoms and inhibiting joint structural damage progression compared to some biologic agents (such as adalimumab) and other JAK inhibitors (such as tofacitinib) in clinical studies, data from clinical trials published in *The New England Journal of Medicine* (NEJM), the World Health Organization (WHO) cost-effectiveness database, national health insurance catalogs (2023), and AbbVie’s official pricing indicate that its cost remains relatively high for most households. This limits its widespread use in low-income regions, and for patients without insurance, the financial burden is particularly severe. The long-term safety management costs and out-of-pocket expenses have a significant impact on low-income patients. In the future, considerations should be given to assistance programs that balance efficacy with long-term costs (Xu et al., 2024; Li et al., 2025).

## 4 Future research directions

Future research can further explore the combination of UPA with Conventional first-line medication, biologic agents, or other small molecule drugs to reduce initial disease activity, control disease progression, and complement the mechanisms of action of different drugs. This approach may reduce the required dosage of a single drug, thus lowering the incidence of side effects. Additionally, personalized treatment is crucial, as clinical manifestations, pathological mechanisms, treatment responses, and prognoses differ significantly among patients. Although treatment options for RA have expanded, a considerable proportion of patients still show inadequate response to standard therapies. Factors such as age, sex, lifestyle, economic status, and drug tolerance influence the choice of treatment and its effectiveness. Personalized therapy allows for the selection of the most appropriate drugs and treatment strategies based on individual patient characteristics. In the future, efforts can be focused on identifying biomarkers that can predict the UPA reaction or adverse reactions, especially those related to cardiovascular or venous thromboembolic risks, thereby reducing such adverse events or reactions. Furthermore, given the positive evidence observed in other disease settings, UPA, as a highly selective JAK1 inhibitor, shows promising therapeutic potential in the management of interstitial lung disease (ILD), particularly RA–associated ILD (RA-ILD). Finally, UPA, by inhibiting JAK pathway signaling, modulates immune responses to alleviate inflammation and associated symptoms.

## 5 Conclusion

UPA, a highly selective JAK1 inhibitor, is a breakthrough tsDMARD for RA. Clinical trials and real-world studies have consistently demonstrated its superior efficacy and treatment persistence compared to adalimumab, MTX monotherapy, abatacept (in bDMARD-IR patients), and other JAK inhibitors. While its high selectivity may reduce off-target toxicities, significant challenges remain. Key concerns include managing safety risks—notably herpes zoster, elevated liver enzymes, and potential long-term MACE—as well as its high cost and the problem of refractory RA. Future directions involve personalized medicine and expanding its application to other diseases like PsA, AS, and ILD. UPA offers a potent oral therapy, but its robust efficacy must be balanced against long-term safety monitoring and affordability.

## Glossary

ACR: American College of Rheumatology
ACR20: American College of Rheumatology 20% improvement criteria
ACR50: American College of Rheumatology 50% improvement criteria
ACR70: American College of Rheumatology 70% improvement criteria
ALT: Alanine Aminotransferase
AST: Aspartate Aminotransferase
AS: Ankylosing Spondylitis
bDMARDs: Biological Disease-Modifying Antirheumatic Drugs
CPK: Creatine Phosphokinase
CDAI: Clinical Disease Activity Index
CI: Confidence Interval
CLOSE-UP: Canadian Long-term Observational Study of Upadacitinib (study name)
CRP: C-reactive Protein
csDMARDs: Conventional Synthetic Disease-Modifying Antirheumatic Drugs
CYC: Cyclophosphamide
DAS28: Disease Activity Score in 28 joints
DMARDs: Disease-Modifying Antirheumatic Drugs
EMA: European Medicines Agency
EULAR: European League Against Rheumatism
FDA: U.S. Food and Drug Administration
HDL: High-Density Lipoprotein
IFN-γ: Interferon-gamma
IL: Interleukin
IL-2: Interleukin-2
IL-3: Interleukin-3
IL-4: Interleukin-4
IL-5: Interleukin-5
IL-6: Interleukin-6
IL-7: Interleukin-7
IL-15: Interleukin-15
IL-21: Interleukin-21
IL-23: Interleukin-23
ILD: Interstitial Lung Disease
JAK: Janus Kinase
JAKi: Janus Kinase inhibitor
JAK-STAT: Janus Kinase–Signal Transducer and Activator of Transcription
LDA: Low Disease Activity
LDL: Low-Density Lipoprotein
MACE: Major Adverse Cardiovascular Events
MAIC: Matching-Adjusted Indirect Comparison
MTX: Methotrexate
NEJM: New England Journal of Medicine
NMSC: Non-Melanoma Skin Cancer
NRI: Non-Responder Imputation
NSAIDs: Non-Steroidal Anti-Inflammatory Drugs
PsA: Psoriatic Arthritis
QoL: Quality of Life
RA: Rheumatoid Arthritis
RA-ILD: Rheumatoid Arthritis–Associated Interstitial Lung Disease
RTX: Rituximab
STAT: Signal Transducer and Activator of Transcription
TEAEs: Treatment-Emergent Adverse Events
TNF: Tumor Necrosis Factor
TNF-α: Tumor Necrosis Factor-alpha
TNFi: Tumor Necrosis Factor inhibitor
tsDMARDs: Targeted Synthetic Disease-Modifying Antirheumatic Drugs
TYK2: Tyrosine Kinase 2
UPA: Upadacitinib
VTE: Venous Thromboembolism
WHO: World Health Organization
